# Induction of IL-9 in Peripheral Lymphocytes of Rheumatoid Arthritis Patients and Healthy Donors by Th17-Inducing Cytokine Conditions

**DOI:** 10.3389/fimmu.2021.668095

**Published:** 2021-04-29

**Authors:** Jana Heim, Giovanni Almanzar, Marc Schmalzing, Michael Gernert, Hans-Peter Tony, Martina Prelog

**Affiliations:** ^1^ Department of Pediatrics, University Hospital Wuerzburg, Wuerzburg, Germany; ^2^ Department of Medicine II, Rheumatology and Clinical Immunology, University Hospital Wuerzburg, Wuerzburg, Germany

**Keywords:** Th9, Th17, interleukin-9, rheumatoid arthritis, PU.1

## Abstract

IL-9-producing Th9 cells display a group of helper T cells with similarities to Th17 and Th2 T cells and have been shown to be involved in synovial inflammation in rheumatoid arthritis (RA) patients. So far, it is unclear which parameters drive Th9 differentiation in lymphocytes derived from RA patients compared to immunologically healthy individuals and whether autocrine mechanisms are able to enhance Th9 polarization. Further, parallel pathways of induction of IL-17-producing cells with Th9 phenotype have to be distinguished from exclusively Th9-inductive mechanisms. Thus, the present study aimed to determine the parameters of Th9 induction by simulation in a standardized inflammatory cytokine milieu.Peripheral naive and non-naive T cells of RA patients and healthy donors (HD) were cultured under Th9 and Th17-driving conditions and phenotypically analyzed by flow cytometry and molecular analysis.Our findings indicate a similar differentiation pathway of Th9 and Th17 cells and similar distributions of IL-9+ T cells in RA and HD regardless of Th9- or Th17-promoting cytokine milieus. Whereas the magnitude and direction of Th9- or Th17-polarization was about the same in RA and HD, IL-17+ CD4+ T cells were significantly stimulated by Th17-inducing conditions in HD. In conclusion, the results indicate that Th9- and Th17-inducing cytokine conditions mimicking autoimmune inflammation in RA may have similar stimulatory effects regarding polarization of peripheral naive and non-naive T cells into Th9 or Th17 cells. The results suggest that the differentiation of Th9 cells may be also induced by Th17-driving conditions.

## Introduction

IL-9-producing Th9 cells have been described as a distinct group of CD4+ helper T cells which are induced by a balanced combination of Interleukin-4 (IL-4) and tumour-growth-factor-beta (TGFβ) (1, 2). The fate of Th9 cell differentiation is controlled by IL-4-receptor-induced up-regulation of signal transducer of transcription 6 (STAT6) and Suppression of Treg-specific transcription factor FoxP3 and of Th2-cell-specific transcription factor GATA-3, whereas TGFβ stimulates the expression of Th9-specific transcription factor PU.1. PU.1 is able to directly bind to the IL-9 promoter and to induce IL-9 production (3). IL-9 receptor (IL-9R) is expressed on naive T cells and thymus-derived natural nTreg, but not on peripherally induced iTreg cells and is relatively low expressed on Th1, Th2 and Th17 cells (4). The role of IL-9 in inflammation has been controversially discussed (5–7). IL-9 has been shown to promote the Th17 differentiation and production of IL-17 and IL-6 (8), in contrast, IL-9 enhances the Suppressive function of Treg (4). An autocrine stimulatory mechanism has been suggested for IL-9-producing Th17 cells which itself express IL-9R.

Plasticity of Th9 cells and their kinship with Th17 and Th2 suggest a role in allergic and autoimmune inflammation. Indeed, high serum levels of IL-9 and soluble IL-9R have been found in rheumatoid arthritis (RA) patients (9, 10). IL-9+ cells could be identified as PU.1-expressing T cells in peripheral blood of chronically-ill RA patients (11) and in synovia tissue of RA patients correlating with inflammatory infiltration (12). IL-9 induced the Th17-differentiation in CD4+ T cells derived from RA patients (13).

So far, it is unclear which parameters drive Th9 differentiation in lymphocytes derived from RA patients compared to immunologically healthy individuals and whether autocrine mechanisms are able to enhance Th9 polarization. Further, parallel pathways of induction of IL-17-producing cells with Th9 phenotype have to be distinguished from exclusively Th9-inductive mechanisms. Thus, the present study aimed to determine the parameters of Th9 induction by simulation in a standardized inflammatory cytokine milieu.

## Methods

### Patients

Peripheral blood mononuclear cells (PBMCs) of 7 ACR/EULAR classification criteria (2010)-defined RA patients and 10 healthy donors (HD) have been obtained by density centrifugation (FicoLite-H, Linaris, Wertheim, Germany) according to standardized laboratory procedures after written informed consent according to the principles of the declaration of Helsinki 2013 and ethical approval of the ethics committee at the University of Wuerzburg (protocol number 239/10). Exclusion criteria for RA and HD were malignoma, monogenetic syndromes, immunodeficiencies, administration of blood products in the past three months, severe infection or allergy requiring medical consultations in the past 8 weeks or vaccinations in the past 4 weeks. RA patients with biologicals or history of biological treatment were excluded. For demographics see **Table 1**.

**Table 1:** Clinical and laboratory characteristics of HD and RA.

	HD	RA
	(n=10)	(n=7)
**General data**		
Age (years), mean (SD)	51.3 (7.2)	59.9 (5.8)
Gender (female), n (%)	9/10 (90%)	4/7 (57.1%)
Disease duration (years), mean (SD)	–	17.1 (11.7)
Joint erosion, n (%)	–	6/7 (85.7%)
**Antibodies**		
elevated RF^a,^ n (%)	–	4/7 (57.1%)
elevated ANA^b^ Titer, n (%)	–	5/7 (71.4%)
elevated anti-CCP^c,^ n (%)	–	4/7 (57.1%)
**Inflammatory laboratory parameters**		
elevated ESR^d,^ n (%)	–	2/7 (28.6%)
elevated CRP^e,^ n (%)	–	4/7 (57.1%)
elevated leukocytes^f,^ n (%)	–	2/7 (28.6%)
**Medication**		
MTX^g^, n (%)	–	7/7 (100%)
Prednisolone^h^, n (%)	–	7/7 (100%)
Leflunomide^i^, n (%)	–	2/7 (28.6%)
Sulfasalazine^j^, n (%)	–	1/7 (14.3%)
Diclofenac^k^, n (%)	–	1/7 (14.3%)
Ibuprofen^l^, n (%)	–	3/7 (42.9%)
Etoricoxib^m^, n (%)	–	1/7 (14.3%)
Metamizole^l^, n (%)	–	1/7 /14.3%)
**Disease activity (DAS 28)**		
Low (0-3.2)	–	3/7 (42.9%)
Moderate (3.2-5.1)	–	2/7 (28.6%)
High (>5.1)	–	2/7 (28.6%)

^a^rheumatoid factor ≥20 IU/mL.

^b^ANA titre ≥ 1:100.

^c^anti-CCP > 7 U/L.

^d^Erythrocyte sedimentation rate >20 mm/h.

^e^C-reactive protein ≥ 0.5 mg/dL.

^f^Leukocytes > 10.200/µL.

^g^Dosage of methotrexate (MTX) 15-25 mg/week, mean 16.4 mg/week.

^h^Dosage of prednisolone 2.5- 20 mg/day, mean 6.5 mg/day.

^i^Dosage of leflunomide 20 mg/day.

^j^Dosage of sulfasalazine 2000 mg/day.

^k^Dosage of diclofenac 75 mg/day.

^l^Dosage of ibuprofen and metamizole as necessary.

^m^Dosage of etoricoxib 90 mg/day.

### Separation of Naive and Non-Naive T Cells and Cell Culture Conditions

PBMCs were separated by two-step magnetic activated cell sorting (MACS) utilizing CD4+ T cell isolation kit and naive CD4+ T cell isolation kit (Miltenyi, Bergisch-Gladbach, Germany) according to the manufacturer’s protocol. Purity of CD4+CD45RA+ naive T cells was >95% as measured by flow cytometry (FACS Canto II, BD, New Jersey, USA) with FACSDiva V6 software (BD). Due to technical efforts to increase the purity of naive T cells for polarization experiments, the purity of non-naive T cells was less (about 50%).

Isolated naive CD4+CD45RA+ and non-naive CD4+CD45R0+ cells (2 x 10^6^ cells/well) were cultivated in RPMI1640 cell culture media (Lonza, Verviers, Belgium) supplemented with 10% fetal calf serum (FCS; Biochrom, Berlin, Germany) and 1% Pen/Strep (Biochrom) together with irradiated (30 Gy) autologous antigen-presenting cells (4 x 10^4^ cells/well). For co-stimulation, anti-CD3/anti-CD28 (1 µg/ml) (Biolegend, San Diego, USA) was added to each well in a 96-well plate. Cells were stimulated by a previously established Th17-inducing cytokine cocktail consisting of recombinant cytokines TGFβ (5 ng/ml) (Biolegend), IL-6 (20 ng/ml) (Immunotools, Friesoythe, Germany), IL-1β (10 ng/ml) (Immunotools) and IL-23 (100 ng/ml) (Biolegend) and by a previously established Th9-inducing cytokine cocktail containing IL-4 (20 ng/ml) (Immunotools), TGFβ (1 ng/ml) (Biolegend), IL-2 (5 ng/ml) (Immunotools) and anti-IFN*γ* (10 µg/ml) (Biolegend) for 3 days at 37°C, 5% CO2 and 95% humidity. The Th9-inducing cytokine cocktail was established in pilot experiments and modified after (1, 14–16).

For simulation of an IL-9 excess, IL-9 (20 ng/ml) (R&D Systems, Minneapolis, USA) was added to Th17- and Th9-inducing cytokine conditions and for inhibition of IL-9 anti-IL-9 (10 µg/ml) (R&D Systems) was added.

### Characterization of Helper T Cells by Flow Cytometry

Resuspension of cells in FACS buffer containing phosphate-buffered saline (PBS) (pH 7.2), 0.5% bovine serum albumin (BSA) and 0.01% NaN_3_ was performed to stain cells with monoclonal antibodies against various cell surface molecules. Monoclonal fluorochrome-labeled antibodies against CD3 were used to identify T cells, CD4 for helper T cells, CD45R0 and CD27 to define differentiation grade of T cells (naive CD45R0-CD27+, memory CD45R0+CD27+, effector CD45R0+CD27-, and effector II or terminally-differentiated-effector-memory T cell re-expressing CD45RA (TEMRA) by CD45R0-CD27-) (17). CCR6 was used as a chemokine receptor characteristic for Th17 cells. CD25+ and CD127- were used as additional markers to identify potential regulatory T cells (Treg) in addition to intracellular FoxP3 staining. Th9 cells were defined as IL-9+ CD4+ helper T cells. IL-9R was applied for identification of IL-9-sensitive cells. For intracellular staining, cells were permeabilized using permeabilization buffer and stained with monoclonal fluorochrome-labelled antibodies against FoxP3 (BD Bioscience) to identify Treg and activated T cells. Ki67 (BD Bioscience) was used to determine proliferating cells. Zombie aqua Dye (Biolegend) was used to exclude dead cells.

Intracellular cytokine production was analyzed following stimulation with phorbol 12-myristate 13-acetate (PMA) (0.03 µg/ml), ionomycin (1 µg/ml) and brefeldin A (10 µg/ml) (all purchased from Sigma-Aldrich, St. Louis, USA) for 4 h at 37°C. Cells were fixed for 20 min at room temperature using fixation buffer (BD, Franklin Lakes, USA) followed by a wash step with permeabilization buffer and stained using monoclonal fluorochrome-labelled antibodies against IL-9, IL-17A, IFN*γ* and IL-10 (all purchased from Biolegend), the latter two cytokines as key cytokines for Th1 and Treg, respectively. Surface and intracellular expression were assessed by flow cytometry (FACSCanto II, BD). Data analysis was performed using FACSDiva software V6 (BD).

### ELISA

In order to analyze IL-9 in the supernatants of cell cultures and in serum samples, an IL-9 ELISA was performed according to the manufacturer’s instructions (eBioscience, San Diego, USA).

### Molecular Analysis

Quantitative PCR for IL-9, IL-9R, IL-17, and PU.1 was performed using designed primers (Eurofins Genomics, Ebersberg, Germany) (for primer sequences see **Supplementary Table 1**). Total RNA was extracted from separated cells applying the RNA Nucleospin isolation kit (Machery Nagel, Düren, Germany) according to the manufacturer’s instructions. Complementary DNA (cDNA) was generated from 1µg RNA using oligodT_18_ primers (Thermo Scientific, Waltham, MA, USA) for reverse transcription with Maxima reverse transcriptase (Thermo Scientific). Real-time PCR was performed using the applied Biosystems^®^ Real Time PCR 7500 (Applied Biosystems, Darmstadt, Germany) utilizing iTaq Universal SYBR green according to the manufacturer’s instructions (Biorad, Ismaning, Germany). Amplification was conducted for 40 cycles. Relative expressions of IL-9, IL-9R, IL-17, and PU.1 were determined by normalizing expression of each gene to β2-microglobulin.

### Statistics

Mann-Whitney U test for not normally distributed independent variables was applied after testing for distribution (Shapiro-Wilks test) with SPSS (Version 24, Chicago, IL). Wilcoxon Rank test was used for not normally distributed dependent variables. Bonferroni’s correction was performed to avoid bias by multiple testing. A p-value <0.05 was considered statistically significant.

## Results

### Increased Switch to TEMRA Phenotype by Th9-Stimulus

In the naive CD4+ T cell fraction, stimulation by unspecific CD3/CD28 stimulation and Th9- and Th17-inducing cytokines, respectively, induced the switch to memory and effector T cell phenotypes as well as to Treg cells (**Supplementary Figure 1** and **Table 2**). Th9-inducing cytokines lead to significantly higher proportions of TEMRA cells compared to Th17-inducing cytokines in the naive CD4+ T cell fraction of HD. In the non-naive CD4+ T cell fraction, memory T cells did not change after Th9- or Th17-inducing cytokines. No significant difference was found between RA and HD.

**Table 2:** Proportions of differentiated T cell subpopulations in native (Pre) and Th9- or Th17-stimulated naive and non-naive CD4+ T cells.

		Naive		Non-naive		
		HD	RA	HD		RA
**Naive**CD45R0-CD27+	Pre	97.8 ± 1.1 (98.1; 95.8- 98.9)	98.5 ± 0.2 (98.4; 98.3 - 98.7)	55.7 ±16.1 (58.6;34.7-73.3)	50.1 ± 10.9 (49.6; 39.5-61.2)	
	Th9	70.0 ±18.7 (71.7; 49.2-90.1)^a^	83.1 ± 12.4 (78.5; 73.6 - 97.1)	25.6 ± 7.8 (28.2; 10.1 - 30.8)^a^	30.1 ± 18.8 (21.0; 17.6 - 51.8)	
	Th17	62.4 ± 24.8 (62.7; 35.8- 89.8)^b^	68.2 ± 25.3 (58.4; 49.3 - 97.0)	25.4 ± 5.5 (26.7; 15.1 - 30.8)^b^	29.6 ± 17.9 (20.2; 18.3 - 50.2)	
**Memory**CD45R0+CD27+	Pre	0.9 ± 0.9 (0.6; 0.3 - 2.7)	0.9 ± 0.4 (0.9; 0.5 - 1.3)	35.0 ± 13.9 (32.7; 17.3- 54.6)	40.1 ± 4.5 (42.6; 34.9 - 42.9)	
	Th9	17.6 ± 13.5 (15.0; 4.5 - 36.5)^a^	10.0 ± 6.9 (13.3; 2.1 - 14.6)	30.1 ± 13.2 (30.7; 7.8 - 49.2)	32.7 ± 15.5 (27.9; 20.1 - 50.0)	
	Th17	31.8 ± 23.1 (33.1; 4.6 - 55.2)^b^	25.3 ± 21.6 (28.9; 2.1 - 44.8)	35.9 ± 16.7 (40.1; 16.3- 55.5)	40.2 ± 8.2 (41.0; 31.7 - 48.0)	
**Effector**CD45R0+CD27-	Pre	0.2 ± 0.3 (0.0; 0.0 - 0.7)	0.1 ± 0.1 (0.0; 0.0 - 0.2)	6.0 ± 3.8 (5.0; 2.1-11.3)	7.7 ± 5.3 (7.3; 2.7 - 13.2)	
	Th9	7.3 ± 5.3 (6.6; 2.0 - 15.0)^a^	4.5 ± 4.3 (4.3; 0.3 - 8.8)	38.7 ± 15.8 (36.2; 20.6-68.2)^a^	33.9 ± 20.1 (27.4; 17.9 - 56.4)	
	Th17	3.8 ± 2.4 (3.2; 1.4 - 7.4)^b^	4.5 ± 4.1 (4.6; 0.4 - 8.6)	33.6 ± 14.7 (28.9; 19.0-58.2)^b^	28.3 ± 10.5 (32.6; 16.4 - 36.0)	
**TEMRA**CD45R0-CD27-	Pre	1.1 ± 0.5 (0.8; 0.8 - 2.0)	0.6 ± 0.3 (0.8; 0.2 - 0.8)	3.3 ± 1.8 (3.1; 1.0 - 6.4)	2.1 ± 2.1 (1.3; 0.5 - 4.4)	
	Th9	5.1 ± 1.8 (5.1; 3.3 - 8.3)^a^	2.5 ± 1.9 (2.6; 0.5 - 4.3)	5.6 ± 4.3 (4.2; 2.3 - 13.9)	3.4 ± 2.3 (2.4; 1.7 - 6.0)	
	Th17	2.0 ± 0.9 (1.6; 1.2 - 3.5)^c^	2.0 ± 1.8 (1.4; 0.6 - 4.1)	5.2 ± 4.1 (3.9; 1.2 - 10.3)	1.9 ± 0.9 (1.7; 1.1 - 2.9)	
**Treg** CD25+CD127-FoxP3+	Pre	0.0 ± 0.0 (0.0; 0.0 - 0.0) ^a,b^	0.0 ± 0.0 (0.0; 0.0 - 0.0)	0.0 ± 0.0 (0.0; 0.0 - 0.1)	0.0 ± 0.0 (0.0; 0.0 - 0.0)	
	Th9	7.6 ± 5.0 (5.9; 2.9 – 14.4)^a^	6.0 ± 3.7 (7.2; 1.9 – 8.9)	7.5 ± 3.9 (6.3; 4.0 – 14.8)^a^	5.2 ± 3.5 (4.4; 2.2 – 9.0)	
	Th17	9.7 ± 3.3 (10.3; 3.6 – 12.9)^b^	7.7 ± 6.0 (8.7; 1.3 – 13.1)	7.0 ± 3.7 (6.0; 3.8 – 13.8)^b^	6.7 ± 3.2 (5.8; 4.1 – 10.3)	

Percentages of naive, memory, effector, TEMRA and Treg cells are given in naive and non-naive CD4+ T cells in native samples pre-stimulation (Pre) and after 3 days culture with Th9- or Th17-inducing cytokines. Values are given in mean ± standard deviation (median; minimum – maximum).

a Differences between Pre and Th9: p = 0.028 (Wilcoxon-rank test).

b Differences between Pre and Th17: p= 0.028 (Wilcoxon-rank test).

c Differences between Th9-inducing and Th17-inducing stimulus: p=0.004 (Mann-Whitney-U-Test).

There were no differences between HD (n=10) and RA (n=7) between each group.

### Similar Effects of Th9- and Th17-Stimulation on Intracellular Cytokine Production, CCR6 Expression, IL-9R Expression and Ki67+ Proliferation

**Figure 1:**
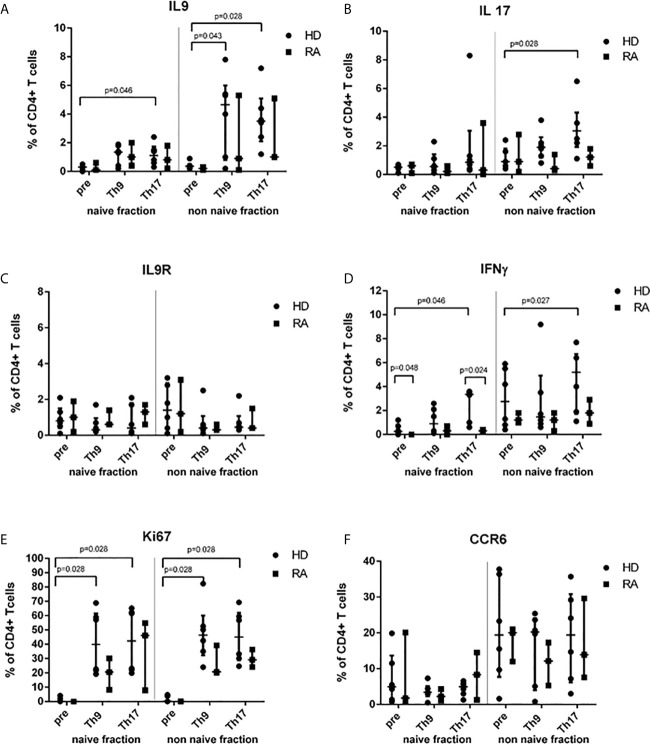
Proportions of cytokine-, IL-9R-, Ki67- or CCR6+ T cells before (pre) stimulation and after 3d Th9- or Th17-stimulation in healthy donors (HD) and rheumatoid arthritis (RA) patients. Percentages of IL-9+ **(A)**, IL-17+ **(B)**, IL-9R+ **(C)**, IFN*γ*+ **(D)**, Ki67+ **(E)** and CCR6+ **(F)** cells are shown in the naive and non-naive fraction of CD4+ peripheral T cells which were investigated before cell culture (pre) and after 3d cell culture under Th9- or Th17-stimulating cytokine conditions in lymphocytes derived from patients with rheumatoid arthritis (RA) and healthy donors (HD) by flow cytometry.

Analysis of intracellular IL-9, IFN*γ*, IL-17 production, of CCR6 expression, IL-9R expression or of Ki67 in separated naive and non-naive CD4+ T cells before cell culture did not show any significant difference between RA and HD except for IFN*γ*-production which was higher in the naive fraction of HD (**Figure 1** and **Supplementary Figure 2**). After 3d cell culture of HD-derived naive CD4+ T cells, proportions of IL-9+ CD4+ T cells were elevated by Th9-stimulus and Th17-stimulus (**Figure 1A**). Despite limitation by moderate purity of separated non-naive T cells, increased proportions of IL-9+ CD4+ T cells were found in both Th9- and Th17-stimulated cultures of HD-derived non-naive CD4+ T cells. Intracellular IL-9 expression was highest in the effector CD4+ T cell subpopulation in switched previously naive CD4+ T cells (**Supplementary Figure 3**).

In contrast to RA, in HD, Th17-stimulus significantly increased the proportion of IL-17+ (**Figure 1B**) and of IFN*γ*+ T cells in CD4+ (**Figure 1D**). This effect of higher IFN*γ* production in HD in differentiated cells of the previously naive T cell fraction was caused by the stimulation of effector CD4+ T cells producing IFN*γ* by Th17-inducing conditions (**Supplementary Figure 3**).

A trend towards down-regulation of IL-9R cell surface expression could be found in the non-naive fraction by Th9- or Th17-stimulus (**Figure 1C**). However, both stimuli increased the Ki67+ proliferation of CD4+ T cells in a similar way in naive and non-naive CD4+ T cells in both HD and RA (**Figure 1E**). Although the CCR6 expression was higher in the non-naive fraction of CD4+ T cells in both HD and RA, CCR6 was neither induced by Th9-stimulus nor by Th17-stimulus (**Figure 1F**).

Proportions of Treg were similarly induced by Th9- and Th17-stimuli in naive CD4+ T cells of HD and of RA (**Table 2**). Production of IL-10 by Treg was not different between Th9- and Th17-inducing stimuli (**Supplementary Figure 3**). However, both stimuli were able to induce IL-17-production in Treg in HD in the naive T cell fraction after cell culture.

### Similar IL-9 Concentrations in Cell-Culture Supernatants After Th9- or Th17-Stimulus in RA and HD

**Figure 2:**
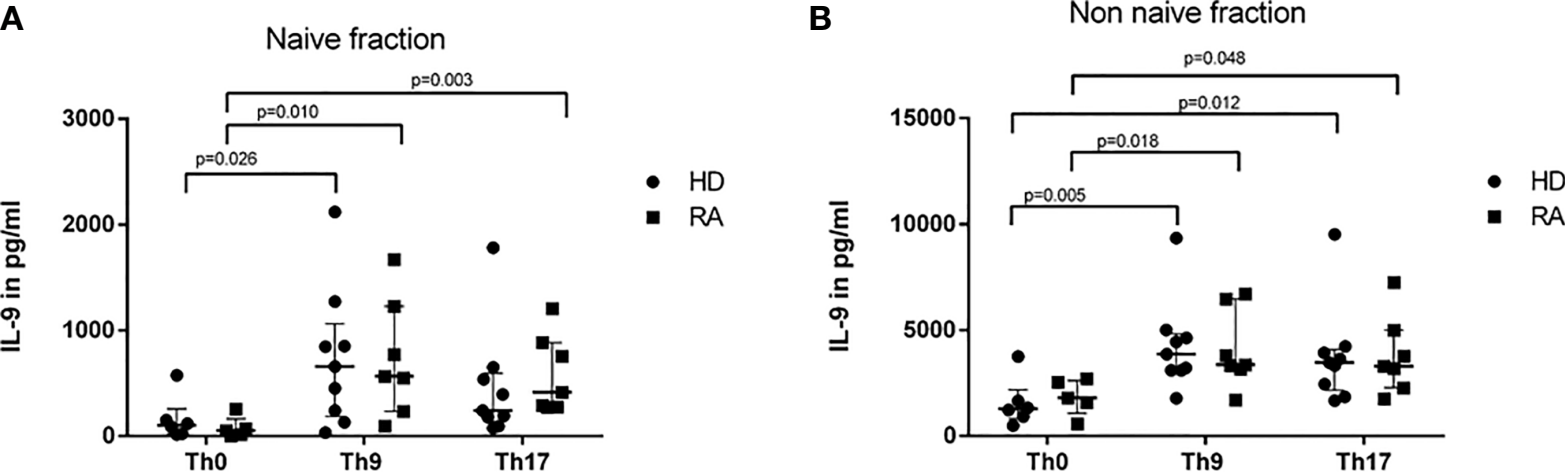
IL-9 concentrations in supernatants of naive **(A)** and non-naive CD4+ cells **(B)** after 3 days cultures with control conditions (Th0) and Th9- or Th17-stimulus in healthy donors (HD) and rheumatoid arthritis (RA) patients. Concentrations of IL-9 were measured in supernatants of naive **(A)** and non-naive CD4+ T cells **(B)** which were culture in control conditions (Th0) and in Th9- or Th17-stimulating cytokine conditions in rheumatoid arthritis (RA) patients and healthy donors (HD) by ELISA.

In order to analyze the overall IL-9 production and release into cell culture media, supernatants after 3d culture of separated naive and non-naive T cells were harvested and IL-9 determined by ELISA (**Figure 2**). Th-9-inducing cytokine conditions lead to a significant increase of IL-9 concentrations in naive CD4+ T cells (**Figure 2A**), whereas in the non-naive T cell group both Th9- and Th17-stimuli drove IL-9 production (**Figure 2B**). In naive CD4+ T cells of RA, the Th17-stimulus significantly induced IL-9 production compared to unspecifically stimulated controls, whereas this difference was not significant in HD.

To adjust for disease activity, results were stratified into low, moderate and high disease activity according to DAS28 (<3.2, 3.2-5.2, >5.1, respectively). Highest IL-9 levels >6000 pg/ml were found in the two RA patients with highly active arthritis, whereas IL-9 concentrations of moderate and low DAS28 activity stayed in the range of concentrations determined in HD (data not shown). One HD had an extreme concentration of IL-9 of 9355 pg/ml without apparent explanation. No association between current medication and IL-9 concentrations was found in RA patients. Serum samples of HD and RA were negative for IL-9.

### Similar PU.1 and IL-9 mRNA Expression After Th9- or Th17-Stimulus in Both RA Patients and HD

Molecular analysis of PU.1 transcription revealed similar values between RA and HD regardless which stimulus was applied (**Table 3**). A significant change towards higher IL-9 mRNA expression could be shown for naive and non-naive CD4+ T cells after 3d culture under Th9- or Th17-stimulating conditions in RA, in HD only for non-naive CD4+ T cells.

**Table 3:** Relative mRNA expression of PU.1, IL-9, IL-17 and IL-9R in control conditions (Th0), Th9- and Th17-inducing conditions in naive and non-naive T cells of healthy donors (HD) and rheumatoid arthritis (RA) patients.

		Naive		Non Naive	
		HD	RA	HD	RA
**PU.1**	Th0	14.6 ± 0.0 (14.6; 14.6-14.6)	13.8 ± 0.4 (13.8; 13.5 – 14.0)	13.1 ± 0.7 (13.5; 12.3-13.6)	12.3 ± 0.2 (12.3; 12.2 – 12.5)
	Th9	14.1 ± 0.3 (14.1; 13.9 - 14.5)	13.9 ± 0.2 (13.9; 13.8 - 14.0)	12.4 ± 0.6 (12.1; 12.0 - 13.0)	13.1 ± 1.1 (13.1; 13.0 - 13.2)
	Th17	13.1 ± 0.8 (13.0; 12.4 - 14.3)	13.8 ± 1.1 (13.7; 12.8 - 15.0)	12.2 ± 0.6 (12.4; 11.4 - 12.8)	12.2 ± 0.5 (12.0; 11.9 - 12.8)
**IL-9**	Th0	8.5 ± 2.4 (9.5; 5.0 – 10.2)	9.4 ± 1.5 (9.9; 7.2 – 10.4)	10.2 ± 3.4 (10.5; 5.8–13.9)	8.6 ± 3.1 (9.0; 4.8 – 11.7)
	Th9	3.8 ± 2.1 (4.0; 1.3 - 5.7)	4.4 ± 1.7 (3.9; 2.8 - 6.8)^a^	2.5 ± 1.5 (2.4; 0.8 - 4.3)^a^	2.3 ± 1.1 (2.6; 0.9 - 3.2)^a^
	Th17	4.9 ± 1.8 (5.8; 2.3 - 5.9)	4.8 ± 1.2 (5.2; 3.0 - 5.8)^b^	4.0 ± 2.2 (4.8; 0.8 - 5.6)^b^	2.8 ± 1.4 (3.3; 0.8 - 3.7)^b^
**IL-17**	Th0	13.4 ± 2.3 (13.4; 11.8-15.0)	12.7 ± 2.0 (12.3; 10.9 – 14.8)	10.7 ± 0.5 (10.8; 10.2-11.1)	9.3 ± 1.9 (9.6; 6.8 – 11.0)
	Th9	16.4 ± 1.1 (16.4; 15.6 - 17.2)	14.7 ± 0.9 (14.6; 13.8 - 15.6)	11.0 ± 1.2 (11.2; 9.3 - 12.2)	10.5 ± 0.7 (10.8; 9.7 - 10.9)
	Th17	12.3 ± 0.3 (12.3; 12.1 - 12.5)	12.9 ± 0.6 (13.1; 12.1 - 13.6)	11.1 ± 1.9 (11.1; 9.0 - 13.3)	10.3 ± 1.1 (10.3; 9.0 - 11.6)
**IL-9R**	Th0	13.2 ± 1.3 (13.1; 11.8-14.7)	13.0 ± 1.6 (12.8; 11.4 – 15.1)	10.5 ± 0.9 (10.6; 9.3–11.5)	10.3 ± 0.9 (10.2; 9.3 – 11.5)
	Th9	11.9 ± 1.3 (11.9; 10.5 - 13.5)	11.6 ± 1.8 (11.2; 9.8 - 14.0)	11.1 ± 0.6 (11.0; 10.3 - 12.0)	11.3 ± 1.1 (11.0; 10.3 - 12.8)
	Th17	12.7 ± 1.0 (12.9; 11.4 - 13.7)	12.7 ± 1.4 (12.4; 11.6 - 14.6)	11.5 ± 0.3 (11.4; 11.2 - 12.0)	12.2 ± 2.0 (11.7; 10.4 - 15.0)

House-keeping gene normalized delta ct values for PU.1, IL-9, IL-17 and IL-9R in four HD and in four RA patients. Values are given in mean ± standard deviation (median; minimum – maximum).

a Differences between Th0 and Th9: p= 0.029 (Mann-Whitney-U-Test).

b Differences between Th0 and Th17: p= 0.029 (Mann-Whitney-U-Test).

Differences between Th9-inducing and Th17-inducing stimulus were not significant. Differences between HD (n=10) and RA (n=7) were not significant.

### No Evidence for Autocrine Control of IL-9 in Separated Naive and Non-Naive CD4+ T Cells in RA and HD

**Figure 3:**
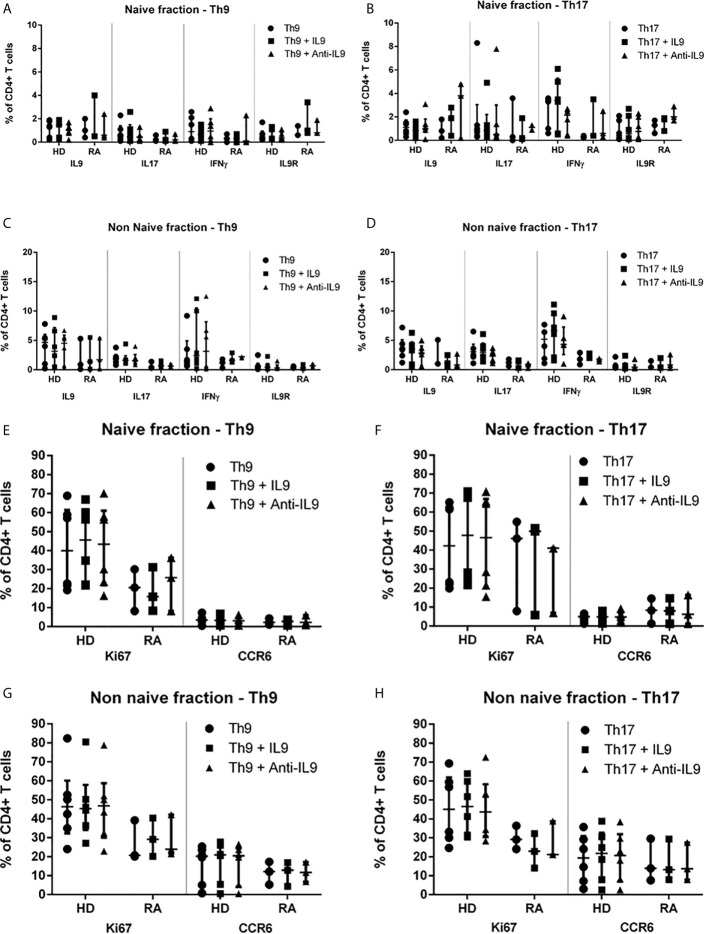
Proportions of IL-9-, IL-17-, IFN*γ*-, IL-9R-, Ki67- and CCR6-expression in naive and non-naive CD4+ T cells in Th9- or Th17-inducing cytokine conditions supplemented with IL-9 or anti-IL-9 in healthy donors (HD) and in rheumatoid arthritis (RA) patients. Percentages of IL-9+, IL-17+, IL-9R+, IFN*γ*+, Ki67+ and CCR6+ cells are shown in the naive of CD4+ peripheral T cells which were investigated after 3d cell culture under Th9- **(A, E)** or Th17-stimulating **(B, F)** cytokine conditions modified by IL-9 excess or anti-IL-9 treatment in lymphocytes derived from patients with rheumatoid arthritis (RA) and healthy donors (HD) by flow cytometry. Percentages of IL-9+, IL-17+, IL-9R+, IFN*γ*+, Ki67+ and CCR6+ cells are shown in the non-naive of CD4+ peripheral T cells which were investigated after 3d cell culture under Th9- **(C, G)** or Th17-stimulating **(D, H)** cytokine conditions modified by IL-9 excess or anti-IL-9 treatment in lymphocytes derived from patients with rheumatoid arthritis (RA) and healthy donors (HD) by flow cytometry.

In order to understand the possible involvement of IL-9-associated autocrine loops which may mitigate our results, an IL-9 excess was applied to Th9- and Th17-stimulated cultures as well as a blockade of IL-9 by a specific antibody (**Supplementary Tables 2** and **3**). Inhibitory function of anti-IL-9 on IL-9 has been previously confirmed by determination of reduction of IL-9 in cell culture supernatants. A concentration of anti-IL-9 was applied leading to 50% inhibition of IL-9 in the Th0 control according to preliminary ELISA results. Neither IL-9 excess nor IL-9-blockade did change the differentiation of naive or non-naive T cell subpopulations in Th9- or Th17-stimulated cultures (**Supplementary Table 2**). Intracellular cytokine production as well as IL-9R (**Figures 3A, B**) or CCR6 cell surface expression and Ki67 proliferation (**Figures 3E, F**) were not significantly influenced by this experimental approach in the naive CD4+ T cell fraction after 3d culture or in the non-naive T cell fraction (**Figures 3C, D, G, H** and **Supplementary Table 3**). mRNA expression of PU.1, IL-9 and IL-9R was not significantly different regarding IL-9 excess or blockade. Also, IL-10 production in Treg was not influenced by IL-9 excess or IL-9 blockade in Th9- or Th17-stimulated cultures (data not shown). In the naive fraction, anti-IL-9 treatment of Th17-stimulated cultures caused a significant reduction of IL-9 concentrations in the supernatants of RA patients compared to HD (**Supplementary Figure 4**). However, a trend to lower IL-9 concentrations secreted by naive or non-naive cells was seen in Th9- and Th17-driving cytokine conditions in both, HD and RA.

## Discussion

The study aimed to assess the induction and characterization of Th9 cells in peripheral CD4+ naive T cells derived from RA patients and HD under standardized Th9- and Th17-inducing cytokine conditions. Our findings indicate a similar differentiation pathway of Th9 and Th17 cells and similar distributions of IL-9+ T cells in RA and HD regardless of Th9- or Th17-promoting cytokine milieus. The induction of IL-9+ CD4+ T cells was more pronounced in the HD group than in RA patients, although the magnitude and direction of Th9- or Th17-polarization was about the same in RA and HD. IL-17+ CD4+ T cells were significantly more stimulated by Th17-inducing conditions in HD. This slight difference between HD and RA may be attributed to unspecific effects of immunosuppressive therapy in RA patients. For IL-9 and IL-17 a significant decline of the serum levels has been shown after 3 months of systemic MTX therapy in patients with psoriasis (18) and furthermore IL-9 serum levels were decreased after treatment with glucocorticoids in patients with systemic lupus erythematosus (19).

Th9 as well as Th17 stimulation with concomitant co-stimulation by anti-CD3/CD28 induced differentiation of naive CD4+ T cells into memory and effector T cells. Th9 appeared to be present in the effector T cell group with a higher differentiation grade into TEMRA cells by Th9-stimulus than by Th17-stimulus. Cells with Treg features, such as FoxP3 expression, are induced by both Th9 and Th17-stimulation and anti-CD3/CD28 with no difference between T cell samples derived from RA and HD. On the one hand a low frequency of Treg cells is suspected to contribute to the pathogenesis of RA (20) on the other hand Tregs are even shown to be enriched in joints of patients with RA (21) which warrants further studies on the function of Tregs in these patients. The impact of IL-9 on the function of Tregs is controversially discussed. A positive (4) as well as a negative (22) effect on Treg function by IL-9 has been shown.

Measurement of the IL-9 concentration in cell culture supernatants revealed an elevation of IL-9 in both HD and RA by Th9- and by Th17-stimulus and at least a trend to reduced secretion of IL-9 by anti-IL-9 treatment *in vitro*. Again, production of IL-9 is equally induced by Th9- or Th17-inducing cytokine conditions in naive and non-naive T cells derived from HD and RA. High IL-9 concentrations were found in supernatants of naive CD4+ T cells derived from two RA patients with high DAS28. A correlation between Th9 cell frequency and DAS28 in patients with RA in both peripheral blood and synovial fluid has been described (13). This may suggest a more pronounced role of Th9 or IL-9 in highly active arthritis in inflamed joint structures. Thus, the approach to stimulate T cells derived from peripheral blood may have overseen effects present in inflamed tissues or under high disease activity. Thus, our results may underestimate the true effect of Th9-inducing stimuli or of IL-9 in autoimmune inflammation in arthritis. However, also one HD presented with high IL-9 concentrations after Th9- or Th17-stimulus for unknown reason.

Whereas the Th9-specific transcription factor PU.1 remains unaffected by Th9- or Th17-inducing cytokines, IL-9 mRNA increased significantly in RA patients and in HD. At the time point of harvesting, proportions of differentiated T cell subpopulations and proportions of cytokine-producing T cells remained unaffected by addition of an IL-9 excess or of 50% inhibitory anti-IL-9 in Th9- or Th17-stimuli-treated cultures of naive or non-naive T cells. Similarly, proliferation (Ki67+) and expression of CCR6 and IL-9R were not altered by this treatment regimen, which excludes the predominance of an IL-9-regulating autocrine mechanism. Upregulation of IL-9R expression is described in affected joints of RA patients (11). One study shows that IL-9 in combination with synovial fluid not only potentiates T cells from synovial fluid of RA patients in producing proinflammatory cytokines but also in expressing higher amounts of IL-9R (13), results that we could not confirm with our data as T cells were derived from peripheral blood. Th9 cells express different chemokine receptors, such as CCR6 and CXCR3, to migrate to autoimmune effector sites. So far known, IL-9 does not have an impact on the chemokine expression on Th9 cells.

Regarding the clinical relevance of IL-9 producing cells, Th9-polarization may not only be associated with pro-inflammatory activities. IL-9-producing type 2 innate lymphoid cells have been shown as the mediators of a molecular and cellular pathway that arranges the resolution of chronic inflammation in RA as evidenced by human and animal studies (23).

Despite the highly standardized cell culture conditions regarding cytokine concentrations, cell numbers and incubation times in this experimental set-up, the interpretation of the results is limited by the case number and the use of peripheral T cells of RA patients who were not naive for immunosuppressive therapies and displayed different disease activity states. However, results from RA patients corresponded very well to the findings in HD samples that IL-9 is induced by both Th9- and Th17-stimulating conditions after unspecific activation with anti-CD3/CD28. Therefore, it seems that IL-9-producing T cells are possibly a “side-product” of the final stretch of polarization of naive or non-naive T cells by either Th9- or Th17-inducing cytokines and corroborates articles that do not emphasize Th9 cells as an individual helper T cell group but subsume them under Th17 or Th2 cells with IL-9-producing features. Our Th9-inducing cytokine cocktail consisted of IL-4, TGFβ, IL-2 and anti-IFN*γ*. However, also other cytokines, such as IL-1, IL-33 (24) and IL-25 (25) may induce Th9 differentiation and should be recognized as Th9-stimulators. The Th17 polarization under Th17-driving conditions was less than expected, which may be due to the decision after performance of pilot experiments to use 3 days incubation for induction of Th9, whereas 5 days would have had the strongest effect on Th17 polarization.

In conclusion, the results indicate that Th9- and Th17-inducing cytokine conditions mimicking autoimmune inflammation in RA may have similar stimulatory effects regarding polarization of peripheral naive and non-naive T cells into Th9 or Th17 cells. The results suggest that the differentiation of Th9 cells may be also induced by Th17-driving conditions.

## Data Availability Statement

The raw data supporting the conclusions of this article will be made available by the authors, without undue reservation.

## Ethics Statement

The studies involving human participants were reviewed and approved by the ethics committee at the University of Wuerzburg, protocol number 239/10. The patients/participants provided their written informed consent to participate in this study.

## Author Contributions

JH: Investigation, formal analysis, writing and editing. GA: Investigation, Methodology, analysis of data, review. MS: Analysis of data, review. MG: Analysis of data, review. H-PT. Analysis of data, review: MP: Conceptualization, methodology, funding acquisition, formal analysis, project administration, visualization, writing original draft, review and editing. All authors contributed to the article and approved the submitted version.

## Funding

The project was funded by an unrestricted grant of the University. This publication was supported by the Open Access Publication Fund of the University of Wuerzburg.

## Conflict of Interest

The authors declare that the research was conducted in the absence of any commercial or financial relationships that could be construed as a potential conflict of interest.

## Supplementary Material

Supplementary Figure 1 Proportions (%) of CD27+ and CD45R0+ T cells in isolated naive and non-naive CD4+ T cells pre-stimulation (Pre) and after 3 days cell culture with Th9- or Th17-stimulus in one representative sample of a healthy donor (HD) and of a rheumatoid arthritis (RA) patient. Percentages indicate the proportion in the respective subpopulation.

Supplementary Figure 2 Proportions (%) of cytokine-, IL-9R-, Ki67- and CCR6-positive T cells in isolated naive and non-naive CD4+ T cells pre-stimulation **(A)** and after 3 days cell culture (**B**, Th9-stimulus; **C**, Th17-stimulus) in one representative sample of a healthy donor (HD) and of a rheumatoid arthritis (RA) patient. Percentages indicate the proportion in the respective subpopulation.

Supplementary Figure 3 Proportions of IL-9-, IL-17-, IL-10-, IFN*γ*- or IL-9R-expressing T cells within differentiated T cells after 3d culture of the naive CD4+ T cell fraction in Th9- or Th17-stimulating cytokine conditions in healthy donors (HD) and rheumatoid arthritis (RA) patients.

Supplementary Figure 4 IL-9 concentrations in supernatants of anti-IL-9-treated cell culture conditions using Th9- or Th17-driving cytokines.
