# Supplementary figures and images for: Induction of IL-9 in Peripheral Lymphocytes of Rheumatoid Arthritis Patients and Healthy Donors by Th17-Inducing Cytokine Conditions
Source: Front Immunol. 2021 Apr 29;12:668095. doi: 10.3389/fimmu.2021.668095 (PMC8117786; doi:10.3389/fimmu.2021.668095)

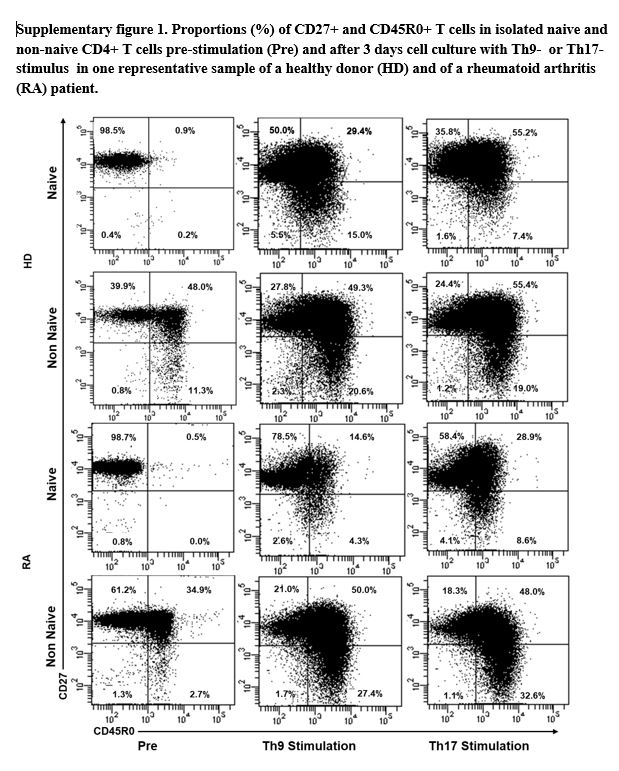

Supplement: Supplementary Figure 1 — Proportions (%) of CD27+ and CD45R0+ T cells in isolated naive and non-naive CD4+ T cells pre-stimulation (Pre) and after 3 days cell culture with Th9- or Th17-stimulus in one representative sample of a healthy donor (HD) and of a rheumatoid arthritis (RA) patient. Percentages indicate the proportion in the respective subpopulation. [file Image_1.jpeg]

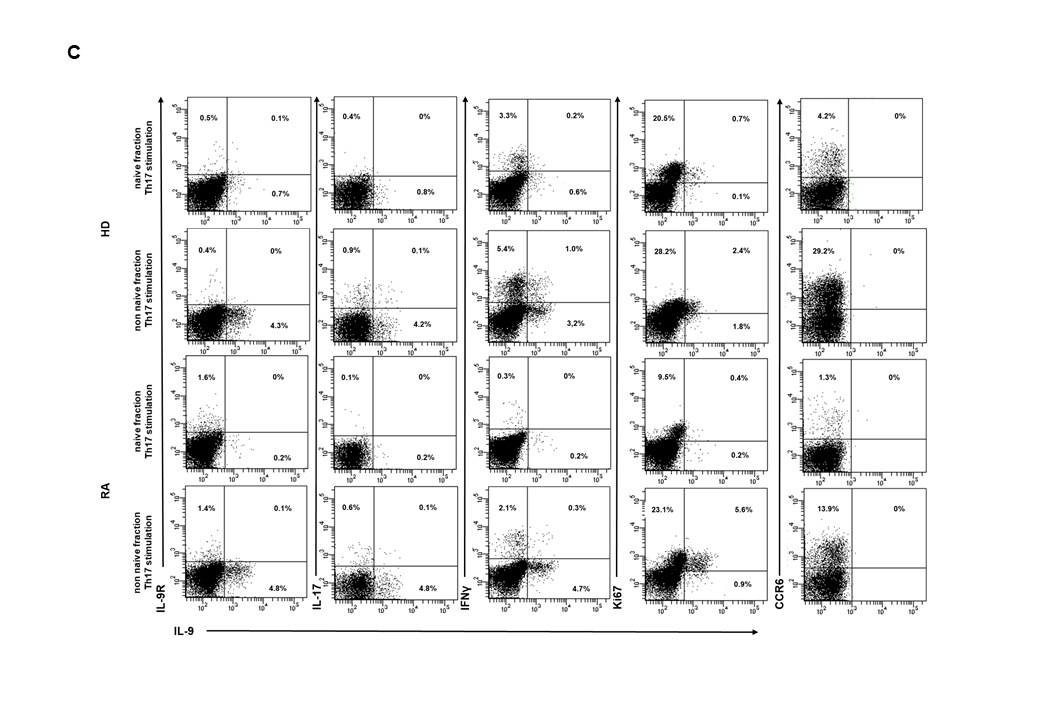

Supplement: Supplementary Figure 2 — Proportions (%) of cytokine-, IL-9R-, Ki67- and CCR6-positive T cells in isolated naive and non-naive CD4+ T cells pre-stimulation (A) and after 3 days cell culture (B, Th9-stimulus; C, Th17-stimulus) in one representative sample of a healthy donor (HD) and of a rheumatoid arthritis (RA) patient. Percentages indicate the proportion in the respective subpopulation. [file Image_2.tif]

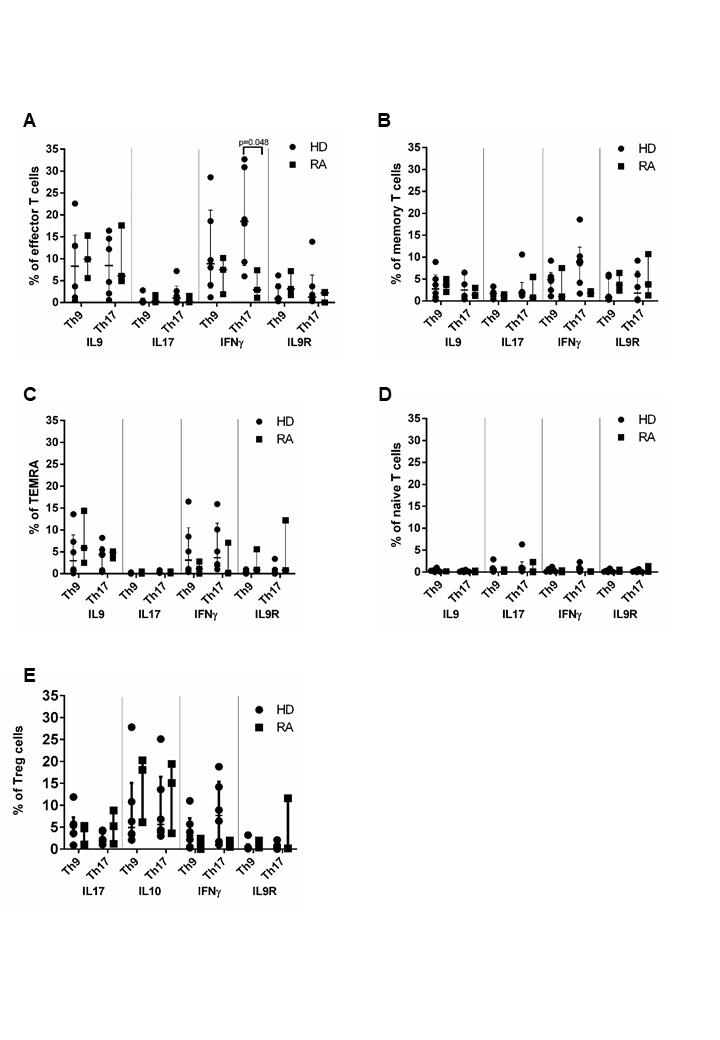

Supplement: Supplementary Figure 3 — Proportions of IL-9-, IL-17-, IL-10-, IFNγ- or IL-9R-expressing T cells within differentiated T cells after 3d culture of the naive CD4+ T cell fraction in Th9- or Th17-stimulating cytokine conditions in healthy donors (HD) and rheumatoid arthritis (RA) patients. [file Image_3.tif]

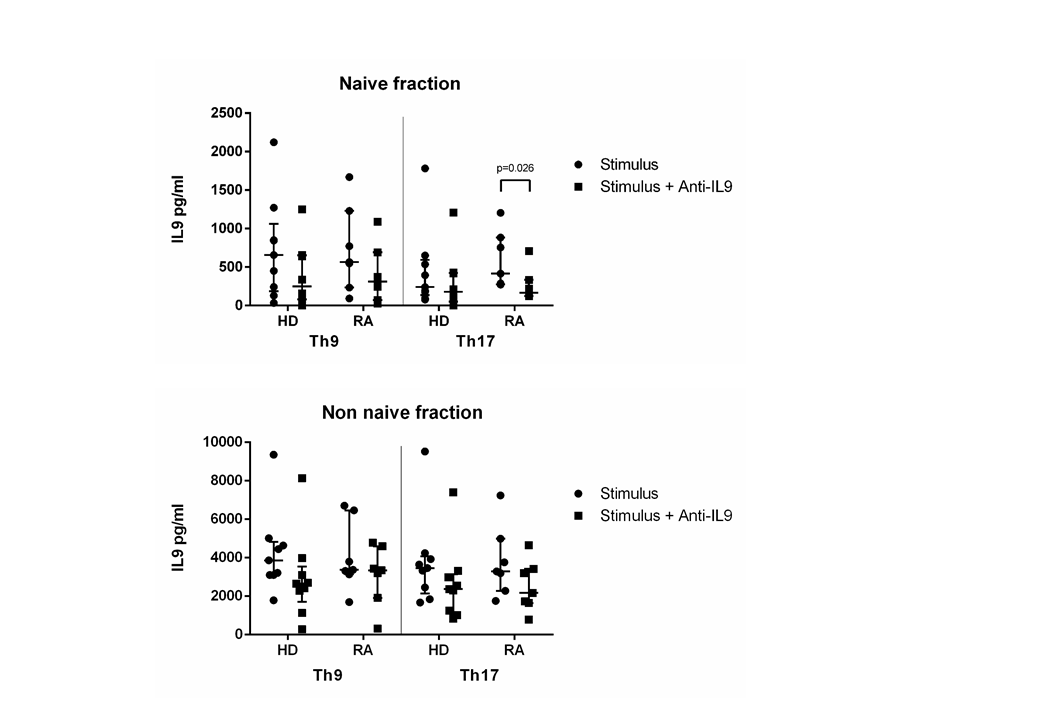

Supplement: Supplementary Figure 4 — IL-9 concentrations in supernatants of anti-IL-9-treated cell culture conditions using Th9- or Th17-driving cytokines. [file Image_4.tif]
