# Supplementary material for: Induction of IL-9 in Peripheral Lymphocytes of Rheumatoid Arthritis Patients and Healthy Donors by Th17-Inducing Cytokine Conditions
Source: Front Immunol. 2021 Apr 29;12:668095. doi: 10.3389/fimmu.2021.668095 (PMC8117786; doi:10.3389/fimmu.2021.668095)
Supplement: Supplementary file 5 [file Table_1.docx]

**Supplementary table 1.**

| **Primer** | **Sequence (5´🡪 3´)** |
| --- | --- |
| β2M F | CCAGCAGAGAATGGAAAGTC |
| β2M R | GATGCTGCTTACATGTCTCG |
| IL9 F | GTGCTAATGTGACCAGTTGTCTC |
| IL9 R | ATCTTGCCTCTCATCCCTCTC |
| IL9R F | CTAGAACCTTCACCTGCCTC |
| IL9R R | GGTCCAGCTTAACGTGTCTC |
| PU.1 F | AGAGCCATAGCGACCATTA |
| PU.1 R | TATCGAGGACGTGCATCT |
| IL17 F | CTCATTGGTGTCACTGCTACTG |
| IL17 R | GGTGGATCGGTTGTAGTAATCTG |

**Legend Supplementary table 1.** The primer sequences are given in forward (F) and reverse (R) directions for beta2-microglobulin (β2M), IL-9, IL-9R, PU.1 and IL-17.

**Supplementary table 2. Proportions of differentiated naive or non-naive T cells in Th9- or Th17-inducing conditions supplemented with IL-9 or anti-IL-9.**

|  | | **Naive** | | **Non Naive** | |
| --- | --- | --- | --- | --- | --- |
|  | | **HD** | **RA** | **HD** | **RA** |
| **Naive**  CD45R0-  CD27+ | Th9 | 70.0 ±18.7 (71.7; 49.2 - 90.1) | 83.1 ± 12.4 (78.5; 73.6- 97.1) | 25.6 ± 7.8 (28.2; 10.1 - 30.8) | 30.1 ± 18.8 (21.0; 17.6 - 51.8) |
|  | +IL9 | 68.6 ± 16.8 (69.9; 48.9 - 88.0) | 81.3 ± 15.9 (82.4; 64.8- 96.6) | 26.3 ± 8.4 (27.8; 10.4 – 34.5) | 30.4 ± 18.2 (22.5; 17.5 – 51.2) |
|  | +Anti-IL9 | 67.1 ± 18.0 (65.8; 47.8 - 89.2) | 79.3 ± 15.6 (74.0; 67.0- 96.9) | 26.8 ± 8.8 (29.2; 9.4 – 33.7) | 29.2 ± 17.6 (20.1; 18.0 – 49.5) |
| **Memory**  CD45R0+  CD27+ | Th9 | 17.6 ± 13.5 (15.0; 4.5 - 36.5) | 10.0 ± 6.9 (13.3; 2.1 - 14.6) | 30.1 ± 13.2 (30.7; 7.8 - 49.2) | 32.7 ± 15.5 (27.9; 20.1 - 50.0 |
|  | +IL9 | 18.4 ± 13.4 (15.6; 5.4 - 37.9) | 10.7 ± 7.6 (11.9; 2.6 - 17.7) | 31.0 ± 15.0 (32.8; 8.5 – 52.7) | 32.9 ± 14.2 (31.6; 19.4 – 47.8) |
|  | +Anti-IL9 | 19.1 ± 15.5 (15.0; 3.6 - 40.5) | 13.4 ± 9.9 (17.3; 2.2 - 20.8) | 27.6 ± 13.6 (28.0; 6.1 – 47.5) | 30.8 ± 15.4 (27.5; 17.4 – 47.6) |
| **Effector**  CD45R0+  CD27- | Th9 | 7.3 ± 5.3 (6.6; 2.0 - 15.0) | 4.5 ± 4.3 (4.3; 0.3 - 8.8) | 38.7 ± 15.8 (36.2; 20.6- 68.2) | 33.9 ± 20.1 (27.4; 17.9 - 56.4) |
|  | +IL9 | 7.3 ± 4.6 (7.5; 2.4 - 14.7) | 5.0 ± 5.4 (3.6; 0.4 - 11.0) | 37.0 ± 16.5 (34.6; 17.7 – 67.4) | 33.5 ± 21.2 (28.3; 15.3 – 56.8) |
|  | +Anti-IL9 | 7.1 ± 4.7 (6.8; 1.9 - 14.0) | 4.8 ± 4.3 (5.5; 0.2 - 8.8) | 38.9 ± 15.6 (35.7; 21.4 – 68.2) | 36.1 ± 19.3 (30.8; 20.1 – 57.5) |
| **TEMRA**  CD45R0-  CD27- | Th9 | 5.1 ± 1.8 (5.1; 3.3 - 8.3) | 2.5 ± 1.9 (2.6; 0.5 - 4.3) | 5.6 ± 4.3 (4.2; 2.3 - 13.9) | 3.4 ± 2.3 (2.4; 1.7 - 6.0) |
|  | +IL9 | 5.8 ± 1.9 (6.7; 2.9 - 7.4) | 3.0 ± 3.1 (2.1; 0.5 - 6.5) | 5.7 ± 4.5 (4.3; 1.8 – 13.7) | 3.1 ± 2.7 (1.8; 1.4 – 6.2) |
|  | +Anti-IL9 | 6.7 ± 4.3 (5.6; 2.1 - 13.9) | 2.4 ± 1.5 (3.2; 0.7 - 3.4) | 6.7 ± 5.1 (5.9; 2.4 – 16.2) | 3.9 ± 2.8 (2.9; 1.6 – 7.1) |
| **Treg**  CD25+  CD127-FoxP3+ | Th9 | 7.6 ± 5.0 (5.9; 2.9 – 14.4) | 6.0 ± 3.7 (7.2; 1.9 – 8.9) | 7.5 ± 3.9 (6.3; 4.0 – 14.8) | 5.2 ± 3.5 (4.4; 2.2 – 9.0) |
|  | +IL9 | 7.4 ± 5.5 (5.9; 1.5 – 15.8) | 5.4 ± 3.3 (3.6; 3.4 – 9.2) | 7.5 ± 5.2 (6.7; 2.5 – 16.8) | 6.2 ± 4.6 (5.0; 2.3 – 11.2) |
|  | +Anti-IL9 | 8.3 ± 4.6 (7.9; 2.1 – 14.5) | 8.6 ± 8.8 (6.2; 1.2 – 18.3) | 9.4 ± 9.7 (5.7; 3.8 – 28.9) | 6.9 ± 4.2 (5.4; 3.7 – 11.6) |
| **Naive**  CD45R0-  CD27+ | Th17 | 62.4 ± 24.8 (62.7; 35.8 - 89.8) | 68.2 ± 25.3 (58.4; 49.3- 97.0) | 25.4 ± 5.5 (26.7; 15.1 - 30.8) | 30.1 ± 18.8 (21.0; 17.6 - 51.8) |
|  | +IL9 | 62.3 ± 21.5 (61.3; 40.5 - 88.0) | 71.6 ± 21.9 (61.5; 56.6- 96.7) | 24.5 ± 6.0 (25.9; 13.3 – 30.6) | 30.1 ± 19.8 (20.0; 17.3 – 52.9) |
|  | +Anti-IL9 | 62.5 ± 21.7 (61.6; 41.7 - 85.6) | 71.2 ± 23.2 (62.7; 53.4- 97.4) | 24.6 ± 6.1 (25.6; 14.2 – 30.6) | 29.1 ± 18.6 (18.6; 18.1 – 50.6) |
| **Memory**  CD45R0+  CD27+ | Th17 | 31.8 ± 23.1 (33.1; 4.6 - 55.2) | 25.3 ± 21.6 (28.9; 2.1 - 44.8) | 35.9 ± 16.7 (40.1; 16.3- 55.5) | 40.2 ± 8.2 (41.0; 31.7 - 48.0) |
|  | +IL9 | 31.2 ± 19.2 (32.7; 6.2 - 52.6) | 22.0 ± 17.8 (25.7; 2.6 - 37.6) | 36.1 ± 18.5 (40.3; 11.9 – 55.1) | 39.4 ± 7.4 (38.5; 32.5 – 47.2) |
|  | +Anti-IL9 | 30.4 ± 20.4 (31.7; 7.7 - 50.8) | 19.7 ± 15.8 (25.9; 1.7 - 31.5) | 34.5 ± 16.5 (35.6; 16.5 – 52.9) | 37.1 ± 7.0 (33.2; 32.8 – 45.2) |
| **Effector**  CD45R0+  CD27- | Th17 | 3.8 ± 2.4 (3.2; 1.4 - 7.4) | 4.5 ± 4.1 (4.6; 0.4 - 8.6) | 33.6 ± 14.7 (28.9; 19.0- 58.2) | 28.3 ± 10.5 (32.6; 16.4 - 36.0) |
|  | +IL9 | 4.2 ± 3.2 (3.2; 1.3 - 10.0) | 4.0 ± 3.7 (4.1; 0.3 - 7.7) | 33.8 ± 16.0 (29.9; 19.8 – 60.4) | 28.5 ± 14.4 (31.5; 12.8 – 41.1) |
|  | +Anti-IL9 | 4.6 ± 2.9 (3.7; 1.9 - 9.9) | 6.9 ± 8.4 (4.0; 0.3 - 16.4) | 35.8 ± 14.7 (31.8; 22.7 – 59.1) | 31.6 ± 15.7 (34.6; 14.6 – 45.5) |
| **TEMRA**  CD45R0-  CD27- | Th17 | 2.0 ± 0.9 (1.6; 1.2 - 3.5) | 2.0 ± 1.8 (1.4; 0.6 - 4.1) | 5.2 ± 4.1 (3.9; 1.2 - 10.3) | 1.9 ± 0.9 (1.7; 1.1 - 2.9) |
|  | +IL9 | 2.3 ± 1.0 (2.0; 1.2 - 3.7) | 2.5 ± 2.4 (1.8; 0.5 - 5.1) | 5.5 ± 5.2 (3.5; 1.2 – 14.5) | 2.0 ± 0.9 (1.8; 1.3 – 3.0) |
|  | +Anti-IL9 | 2.5 ± 1.0 (2.4; 1.5 - 3.8) | 2.2 ± 1.9 (1.7; 0.7 - 4.3) | 5.2 ± 3.6 (4.9; 1.6 – 10.2) | 2.3 ± 1.2 (1.7; 1.5 – 3.6) |
| **Treg**  CD25+  CD127-FoxP3+ | Th17 | 9.7 ± 3.3 (10.3; 3.6 – 12.9) | 7.7 ± 6.0 (8.7; 1.3 – 13.1) | 7.0 ± 3.7 (6.0; 3.8 – 13.8) | 6.7 ± 3.2 (5.8; 4.1 – 10.3) |
|  | +IL9 | 10.9 ± 3.1 (10.4; 7.9 – 15.0) | 13.7 ± 9.5 (17.1; 3.0 – 21.0) | 7.9 ± 4.4 (6.8; 2.9 – 15.5) | 6.5 ± 5.6 (3.5; 3.1 – 13.0) |
|  | +Anti-IL9 | 7.5 ± 4.3 (6.8; 2.3 – 14.2) | 13.9 ± 10.6 (17.9; 1.9 – 21.9) | 5.7 ± 2.6 (5.8; 1.8 – 8.3) | 6.2 ± 4.6 (5.0; 2.3 – 11.2) |

**Legend Supplementary table 2.** Percentages of naive, memory, effector, TEMRA and Treg cells are given in naive and non-naive CD4+ T cells after culture with Th9- or Th17-inducing cytokines and supplemented with IL-9 or anti-IL-9. Values are given in mean ± standard deviation (median; minimum–maximum). Differences between Th9- or Th17 stimulated cells and supplementation with IL9 or anti-IL9 were not significant.

**Supplementary table 3. Proportions of cytokine-, IL-9R-, Ki67 or CCR6-positive naive or non-naive T cells in Th9- or Th17-inducing conditions supplemented with IL-9 or anti-IL-9.**

|  |  | **Naive** | | **Non Naive** | |
| --- | --- | --- | --- | --- | --- |
|  |  | **HD** | **RA** | **HD** | **RA** |
| **IL-9** | **Th9** | 1.2 ± 0.7 (1.4; 0.2 - 1.9) | 1.1 ± 0.8 (1.0; 0.4 - 2.0) | 4.0 ± 2.9 (4.7; 0.2 - 7.8) | 2.1 ± 2.8 (0.9; 0.1 - 5.3) |
|  | **+IL-9** | 1.3 ± 0.7 (1.5; 0.3 - 1.9) | 1.7 ± 2.0 (0.5; 0.5 - 4.0) | 3.7 ± 3.4 (1.4; 0.3 – 8.9) | 2.3 ± 2.8 (1.4; 0.1 - 5.5) |
|  | **+Anti-IL-9** | 1.0 ± 0.5 (1.1; 0.2 - 1.7) | 1.1 ± 1.1 (0.6; 0.4 - 2.4) | 3.7 ± 2.7 (4.5; 0.3 – 6.7) | 2.4 ± 2.7 (1.7; 0.1 – 5.4) |
| **IL-17** | **Th9** | 0.8 ± 0.8 (0.6; 0.1 - 2.3) | 0.3 ± 0.3 (0.2; 0.1 - 0.6) | 2.0 ± 1.0 (1.9; 0.8 - 3.8) | 0.7 ± 0.6 (0.4; 0.3 - 1.4) |
|  | **+IL-9** | 0.8 ± 0.9 (0.5; 0.1 - 2.6) | 0.4 ± 0.4 (0.2; 0.1 - 0.9) | 2.0 ± 1.2 (1.6; 1.1 - 4.4) | 0.9 ± 0.5 (0.7; 0.5 – 1.5) |
|  | **+Anti-IL-9** | 0.6 ± 0.6 (0.5; 0.1 - 1.3) | 0.3 ± 0.3 (0.2; 0.1 - 0.7) | 1.9 ± 1.1 (1.6; 0.9 – 4.0) | 07 ± 0.3 (0.6; 0.5 – 1.0) |
| **IFNγ** | **Th9** | 1.2 ± 1.1 (0.9; 0.1 – 2.6) | 0.3 ± 0.4 (0.3; 0.0 – 0.7) | 2.9 ± 3.3 (1.5; 0.6 – 9.2) | 1.1 ± 0.8 (1.2; 0.3 – 1.8) |
|  | **+IL-9** | 0.8 ± 0.6 (0.7; 0.1 – 1.5) | 0.3 ± 0.4 (0.3; 0.0 – 0.7) | 4.7 ± 5.0 (2.4; 0.2 – 12.1) | 1.9 ± 0.9 (1.6; 1.3 – 2.9) |
|  | **+Anti-IL-9** | 1.1 ± 1.1 (1.0; 0.0 – 2.9) | 0.8 ± 1.3 (0.1; 0.0 – 2.3) | 4.3 ± 4.9 (3.2; 0.1 – 12.5) | 2.1 ± 0.1 (2.2; 2.0 – 2.2) |
| **IL-9R** | **Th9** | 0.6 ± 0.6 (0.3; 0.2 - 1.7) | 0.9 ± 0.5 (0.6; 0.6 - 1.4) | 0.7 ± 0.9 (0.4; 0.1 - 2.5) | 0.4 ± 0.2 (0.3; 0.3 - 0.6) |
|  | **+IL-9** | 0.6 ± 0.5 (0.3; 0.3 - 1.3) | 1.7 ± 1.4 (1.0; 0.8 - 3.4) | 0.7 ± 0.8 (0.4; 0.1 – 2.3) | 0.5 ± 0.4 (0.3; 0.2 – 0.9) |
|  | **+Anti-IL-9** | 0.5 ± 0.3 (0.4; 0.2 - 1.1) | 1.2 ± 0.6 (0.8; 0.8 - 1.9) | 0.5 ± 0.5 (0.3; 0.2 – 1.5) | 0.7 ± 0.3 (0.7; 0.4 – 1.0) |
| **Ki67** | **Th9** | 41.4 ± 22.5 (39.9; 19.2 - 68.9) | 19.6 ± 11.0 (20.5; 8.2 - 30.2) | 47.8 ± 19.9 (46.4; 24 - 82.4) | 26.7 ± 10.9 (20.7; 20.1 - 39.2) |
|  | **+IL-9** | 43.7 ± 20.0 (45.6; 21.7 - 66.9) | 18.5 ± 11.7 (15.7; 8.4 - 31.3) | 47.4 ± 18.1 (45.3; 27.0 – 80.4) | 29.9 ± 10.1 (29.1; 20.2 – 40.4) |
|  | **+Anti-IL-9** | 42.5 ± 21.9 (43.4; 16.3 - 70.2) | 23.4 ± 14.2 (25.8; 8.2 - 36.3) | 46.6 ± 19.2 (46.8; 22.9 – 78.8) | 29.2 ± 11.3 (23.9; 21.5 – 42.1) |
| **CCR6** | **Th9** | 3.7 ± 2.2 (3.4; 0.5 - 7.3) | 2.5 ± 1.6 (2.2; 1.1 - 4.3) | 15.9 ± 10.3 (20.2; 0.8 - 25.4) | 11.6 ± 6.0 (12.1; 5.3 - 17.3) |
|  | **+IL-9** | 3.5 ± 2.1 (3.3; 0.6 - 6.8) | 2.4 ± 1.5 (2.7; 0.7 - 3.7) | 16.9 ± 11.3 (20.9; 0.5 – 27.7) | 11.4 ± 6.4 (12.9; 4.4 – 16.9) |
|  | **+Anti-IL-9** | 3.2 ± 1.9 (3.1; 0.7 - 6.2) | 3.0 ± 2.7 (2.2; 0.9 - 6.0) | 16.2 ± 10.6 (20.5; 0.5 – 26.6) | 11.9 ± 5.2 (11.7; 6.8 – 17.1) |
| **IL-9** | **Th17** | 1.2 ± 0.8 (1.1; 0.3 - 2.4) | 0.9 ± 0.8 (0.8; 0.2 - 1.8) | 3.7 ± 2.0 (3.5; 1.2 - 7.2) | 2.4 ± 2.4 (1.0; 1.0 - 5.1) |
|  | **+IL-9** | 0.9 ± 0.5 (0.9; 0.2 - 1.6) | 1.7 ± 1.2 (1.9; 0.4 - 2.8) | 3.2 ± 2.2 (3.6; 0.5 – 6.3) | 1.4 ± 1.0 (1.1; 0.6 – 2.5) |
|  | **+Anti-Il-9** | 1.3 ± 1.0 (1.0; 0.1 - 3.1) | 2.9 ± 2.4 (3.8; 0.2 - 4.8) | 2.6 ± 1.7 (2.9; 0.6 – 5.0) | 1.4 ± 1.2 (0.8; 0.6 – 2.7) |
| **IL-17** | **Th17** | 2.0 ± 3.1 (0.9; 0.3 - 8.3) | 1.3 ± 2.0 (0.3; 0.0 - 3.6) | 3.3 ± 1.8 (3.1; 1.1 - 6.5) | 1.2 ± 0.6 (1.2; 0.6 - 1.8) |
|  | **+IL-9** | 1.5 ± 1.7 (0.9; 0.3 - 4.9) | 0.7 ± 1.0 (0.2; 0.1 - 1.9) | 3.2 ± 1.7 (2.9; 1.4 – 6.1) | 1.1 ± 0.7 (1.4; 0.3 – 1.6) |
|  | **+Anti-Il-9** | 1.8 ± 3.0 (0.5; 0.2 - 7.8) | 0.7 ± 0.7 (0.9; 0.0 - 1.3) | 2.3 ± 1.0 (2.3; 1.1 – 3.7) | 0.8 ± 0.4 (0.9; 0.3 – 1.1) |
| **IFNγ** | **Th17** | 2.6 ± 1.4 (3.4; 0.6 – 3.6) * | 0.3 ± 0.1 (0.3; 0.2 – 0.4) * | 4.6 ± 2.7 (5.2; 1.1 – 7.7) | 1.9 ± 1.0 (1.8; 0.9 – 2.9) |
|  | **+IL-9** | 3.2 ± 2.3 (3.4; 0.5 – 6.1) | 1.4 ± 1.8 (0.4; 0.4 – 3.5) | 6.3 ± 3.9 (6.7; 1.6 – 11.1) | 2.3 ± 0.5 (2.0; 2.0 – 2.8) |
|  | **+Anti-Il-9** | 1.7 ± 1.0 (2.2; 0.4 – 2.7) | 1.1 ± 1.2(0.6; 0.3 – 2.5) | 4.8 ± 2.9 (4.3; 1.0 – 9.1) | 1.7 ± 0.3 (1.8; 1.4 – 1.9) |
| **IL-9R** | **Th17** | 0.8 ± 0.9 (0.4; 0.1 - 2.1) | 1.2 ± 0.6 (1.3; 0.6 - 1.7) | 0.8 ± 0.7 (0.5; 0.3 - 2.2) | 0.8 ± 0.6 (0.4; 0.4 - 1.5) |
|  | **+IL-9** | 1.0 ± 1.1 (0.6; 0.1 - 2.7) | 1.5 ± 0.6 (1.8; 0.8 - 1.9) | 0.7 ± 0.9 (0.4; 0.2 – 2.4) | 0.9 ± 1.0 (0.4; 0.3 – 2.0) |
|  | **+Anti-Il-9** | 1.0 ± 1.0 (0.8; 0.1 - 2.3) | 2.2 ± 0.6 (2.0; 1.7 - 2.9) | 0.6 ± 0.6 (0.5; 0.2 – 1.8) | 1.2 ± 1.2 (0.8; 0.3 – 2.6) |
| **Ki67** | **Th17** | 42.4 ± 22.7 (42.3; 19.9 - 65.2) | 36.3 ± 25.0 (46.1; 7.9 - 54.9) | 45.6 ± 18.5 (45; 24.7 -69.3) | 29.9 ± 6.2 (29.1; 24.1 - 36.4) |
|  | **+IL-9** | 46.7 ± 24.5 (47.9; 21.7 - 70.9) | 35.8 ± 26.0 (49.9; 5.8 - 51.6) | 46.5 ± 14.3 (46.5; 30.5 – 63.9) | 23.0 ± 9.1 (22.9; 14.0 – 32.2) |
|  | **+Anti-IL-9** | 44.4 ± 25.2 (46.6; 15.5 - 70.8) | 29.7 ± 19.7 (41.0; 7.0 - 41.2) | 45.6 ± 17.1 (43.7; 28.4 – 72.6) | 27.0 ± 10.1 (21.2; 21.1 – 38.7) |
| **CCR6** | **Th17** | 4.5 ± 2.0 (5.0; 1.3 - 6.6) | 8.0 ± 6.6 (8.3; 1.3 - 14.5) | 19.0 ± 12.8 (19.4; 3 -35.7) | 17.0 ± 11.3 (13.9; 7.6 - 29.6) |
|  | **+IL-9** | 4.8 ± 2.3 (4.9; 1.4 - 8.0) | 8.0 ± 6.8 (8.0; 1.2 - 14.7) | 20.6 ± 13.7 (21.8; 2.5 – 38.8) | 16.9 ± 11.2 (13.2; 8.0 – 29.4) |
|  | **+Anti-IL-9** | 5.0 ± 2.6 (4.8; 1.9 - 9.2) | 7.9 ± 7.7 (6.2; 1.2 - 16.4) | 20.1 ± 13.3 (20.7; 2.6 – 38.4) | 16.4 ± 10.1 (13.7; 7.9 – 27.5) |

**Legend Supplementary table 3.** Percentages of IL-9-, IL-17-, IFNγ-, IL-9R-, Ki67- and CCR6- positive T cells are given in naive and non-naive CD4+ T cells after culture with Th9- or Th17-inducing cytokines and supplemented with IL-9 or anti-IL-9. Values are given in mean ± standard deviation (median; minimum–maximum). * Differences between HD and RA: p=0.024 (Mann-Whitney-U-Test).
